# Supplementary material for: Endoplasmic reticulum stress enhances fibrosis through IRE1α‐mediated degradation of miR‐150 and XBP‐1 splicing
Source: EMBO Mol Med. 2016 May 25;8(7):729–44. doi: 10.15252/emmm.201505925 (PMC4931288; doi:10.15252/emmm.201505925)
Supplement: Supplementary file 1 — Expanded View Figures PDF [file EMMM-8-729-s001.pdf]

Expanded View Figures

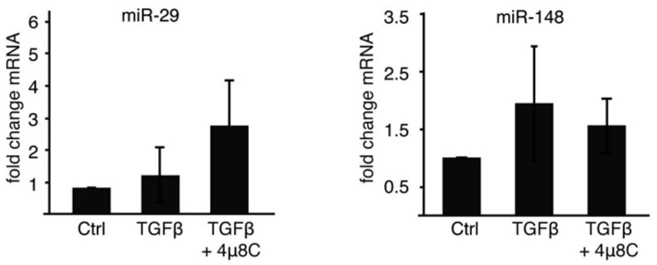

Figure EV1. The levels of miR-29 and miR-148 are not affected by treatment with 4μ8C.
Error bars indicate s.e.m. *n* = 4.

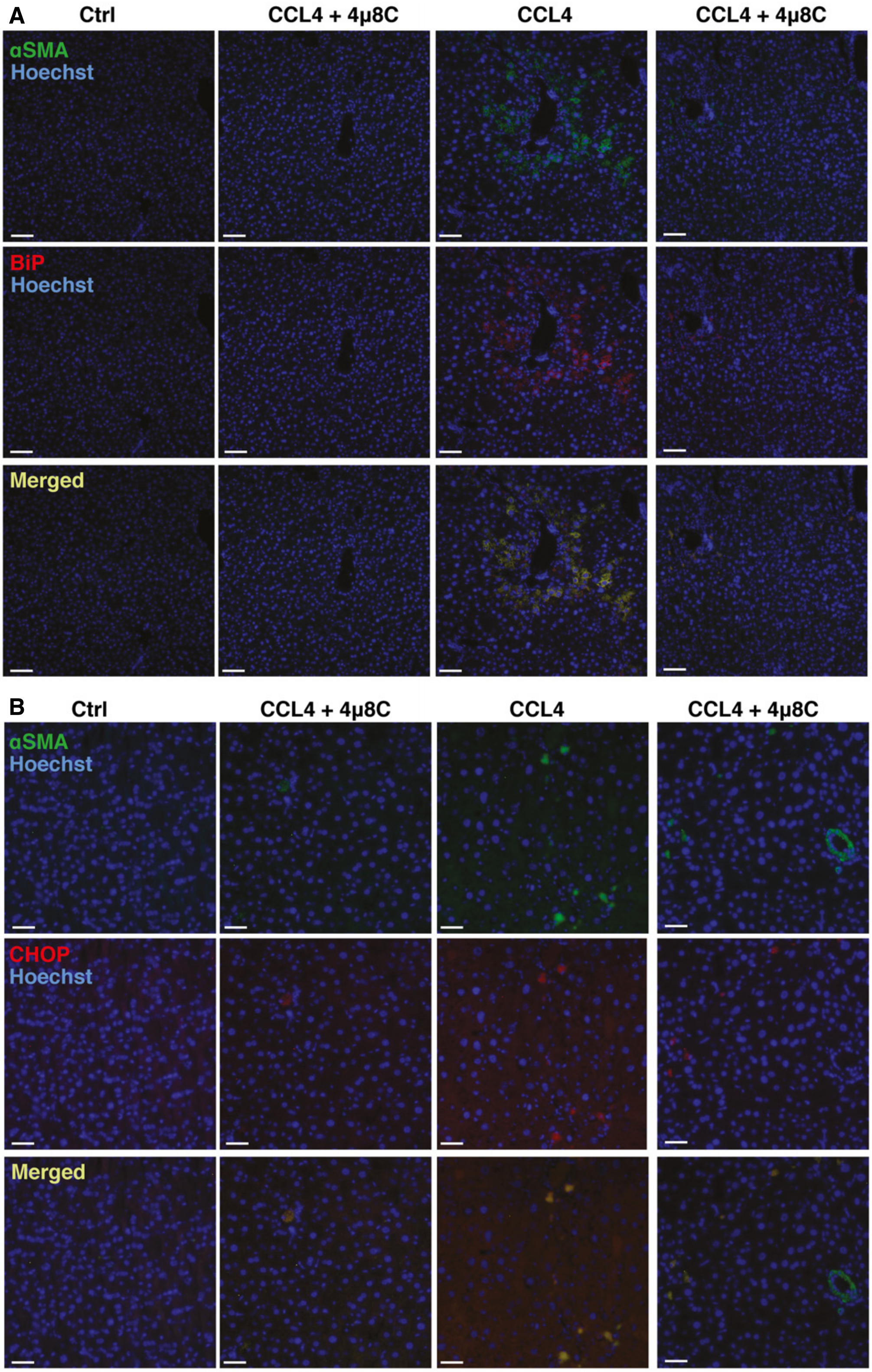

Figure EV2. Activated hepatic stellate cells express UPR markers during liver fibrosis.

A Representative images of CCl_4-induced cirrhotic livers from C57BL/6 mice. Livers were co-stained with anti-αSMA and anti-BiP antibodies. Scale bars = 50 μm.

B Representative images of livers co-stained with anti-αSMA and anti-CHOP antibodies. Scale bars = 50 μm.

 © 2016 The Authors

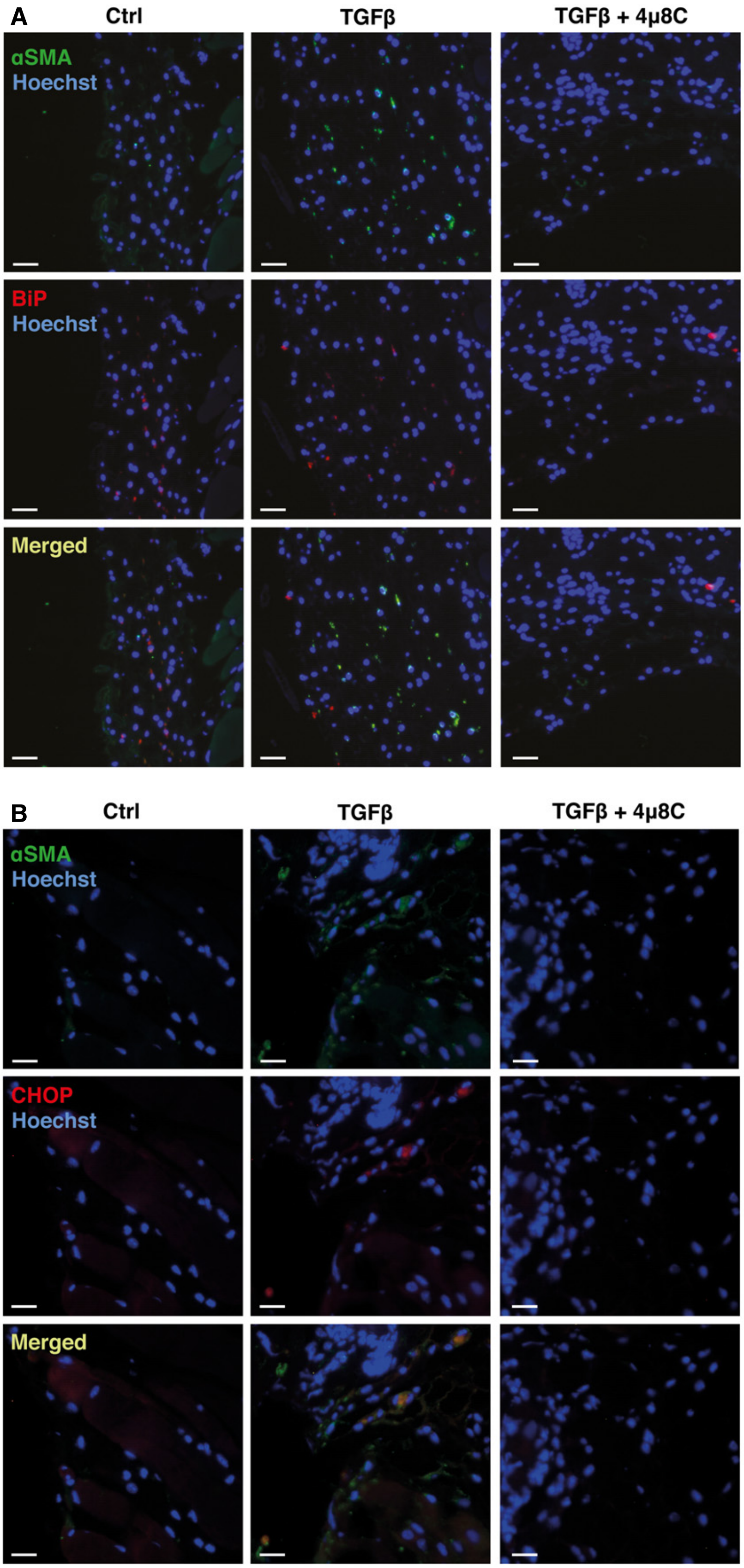

Figure EV3. Myofibroblasts express UPR markers during skin fibrosis.

- A Representative images of skin from control or 4μ8C-treated C57BL/6 mice with TGFβ-induced skin fibrosis. Sections of skin were co-stained with anti-αSMA and anti-BiP antibodies. Scale bars = 50 μm.
- B Representative images of mouse skin co-stained with anti-αSMA and anti-CHOP antibodies. Scale bars = 50 μm.

© 2016 The Authors
