# Supplementary material for: Endoplasmic reticulum stress enhances fibrosis through IRE1α‐mediated degradation of miR‐150 and XBP‐1 splicing
Source: EMBO Mol Med. 2016 May 25;8(7):729–44. doi: 10.15252/emmm.201505925 (PMC4931288; doi:10.15252/emmm.201505925)
Supplement: Supplementary file 2 — Table EV1 [file EMMM-8-729-s002.docx]

**Table EV1**

Pre-miR-150 and XBP-1 mini stem-loop sequences.

PremiR150 5’CUCCCCAUGGCCCUGUCUCCCAACCCUUGUACCAGUGCUGGGCUCAGACCCUGGUACAGGCCUGGGGGACAGGGACCUGGGGAC-3’

XBP-1 mini stem-loop

5’-CUGAGUCCGCAGCACUCAG-3’
