# Supplementary material for: Endoplasmic reticulum stress enhances fibrosis through IRE1α‐mediated degradation of miR‐150 and XBP‐1 splicing
Source: EMBO Mol Med. 2016 May 25;8(7):729–44. doi: 10.15252/emmm.201505925 (PMC4931288; doi:10.15252/emmm.201505925)
Supplement: Supplementary file 3 — Table EV2 [file EMMM-8-729-s003.docx]

**Table EV2**

Primer sequences and antibodies used.

Primers

18S FW : 5’-AGTCCCTGCCCTTTGTACACA-3’

18S REV : 5’-GATCCGAGGGCCTCACTAAAC-3’

β-actin FW : 5’- tctacaatgagctgcgtgtg-3’

β-actin REV : 5’- agcctggatagcaacgtaca-3’

hsa - αSMA FW : 5’- TTCAATGTCCCAGCCATGTA-3’

hsa - αSMA REV : 5’- GCAAGGCATACCCTCATAG-3’

mmu- αSMA FW : 5’- CTGACAGAGGCACCACTGAA-3’

mmu- αSMA REV : 5’- CATCTCCAGAGTCCAGCACA-3’

XBP-1 FW for splicing: 5’ –AAACAGAGTAGCAGCTCAGACTGC-3'

XBP-1 REV for splicing: 5’- TCCTTCTGGGTAGACCTCTGGGAG-3’

XBP-1 FW (for qPCR) : 5’-CCTGCTGCAGAGGTGCACGTAG-3’

XBP-1 REV (for qPCR) : 5’-CCTGCACCTGCTGCGGACTC-3’

IRE1a FW : 5’-CCGAACGTGATCCGCTACTTCT-3’

IRE1a REV : 5’-CGCAAAGTCCTTCTGCTCCACA-3’

Collagen 1α2 FW : 5’- GGCCCTCAAGGTTTCCAAGG-3’

Collagen 1α2 REV : 5’- CACCCTGTGGTCCAACAACTC-3’

TBP FW : 5´-AGTGACCCAGCATCACTGTTT-3´

TBP REV: 5´-GGCAAACCAGAAACCCTTGC-3

CTGF FW: 5´-GACCTGGAAGAGAACATTAAGAAGG-3´

CTGF REV: 5´-TCGGTATGTCTTCATGCTGGTG-3´

Collagen1α2 (SSc) FW 5´-TGCTTGCAGTAACCTTATGCCTA-3´

Collagen1α2 (SSc) REV: 5 ´-CAGCAAAGTTCCCACCGAGA-3´

miR-150, SNORD95, SNORD68 : Qiagen product number : MS00003577, MS00033712 and MS00033726 respectively.

Antibodies

β-actin: Abcam AC-15 ab6276

αSMA (WB): Abcam E-184 ab32575

αSMA (IHC): Sigma A2547

cMYB: Santa Cruz Biotechnology C-19 sc-517

BiP: Abcam, ab21685,

CHOP (WB) : Abcam 9C8 ab11419

CHOP (IHC): Santa Cruz, sc575
