# Supplementary figures and images for: Endoplasmic reticulum stress enhances fibrosis through IRE1α‐mediated degradation of miR‐150 and XBP‐1 splicing
Source: EMBO Mol Med. 2016 May 25;8(7):729–44. doi: 10.15252/emmm.201505925 (PMC4931288; doi:10.15252/emmm.201505925)

Source data Figure 1D

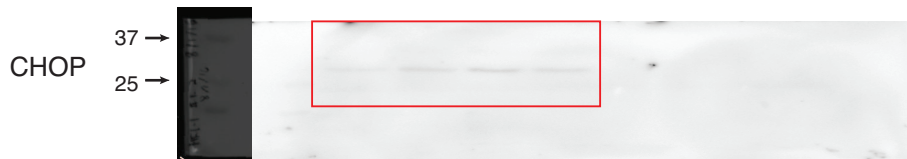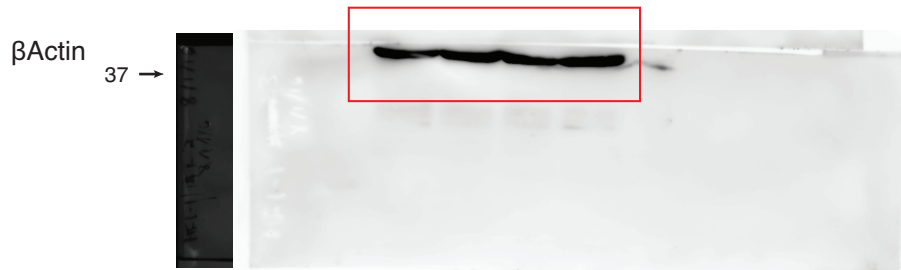

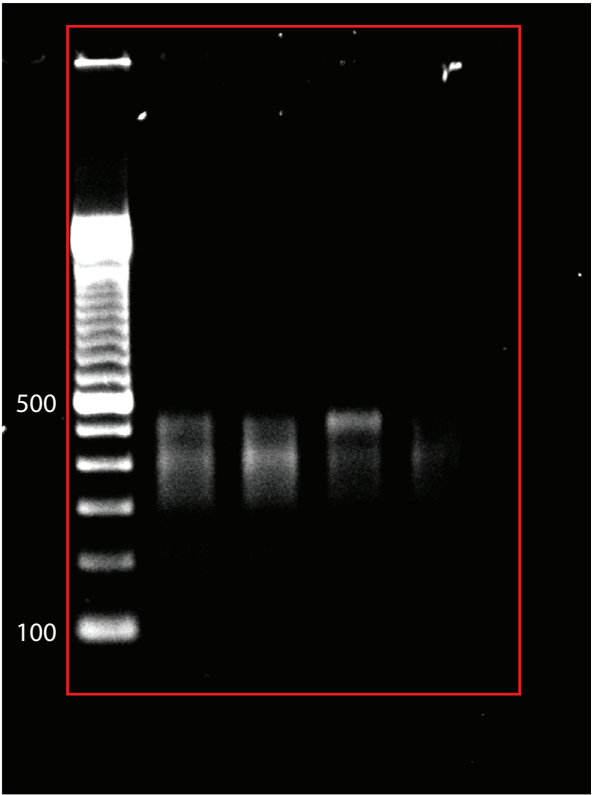

Supplement: Supplementary file 5 — Source Data for Figure 1 [file EMMM-8-729-s004.pdf]

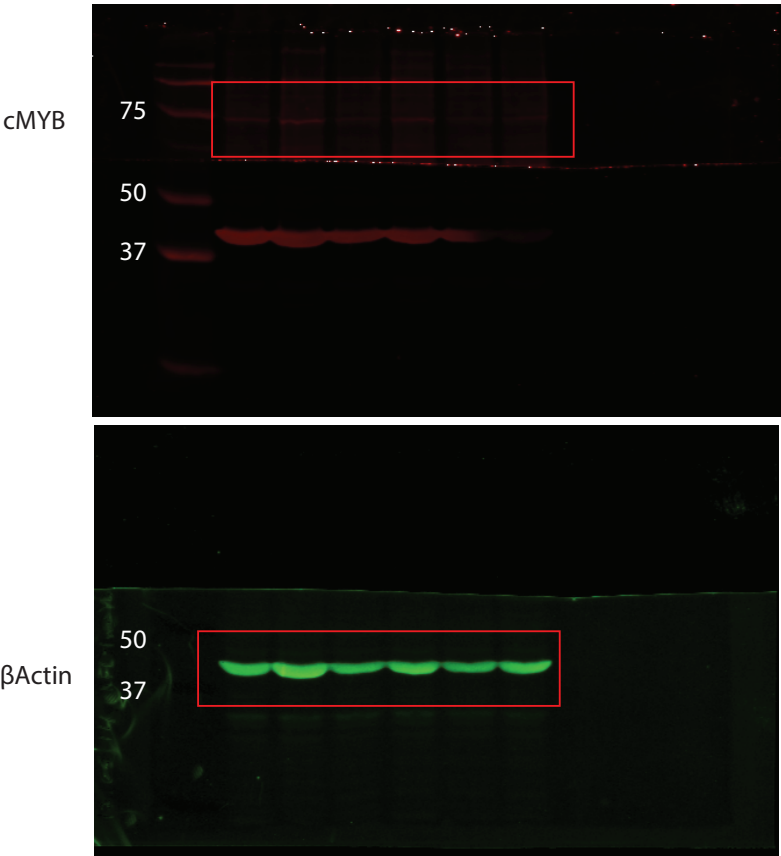

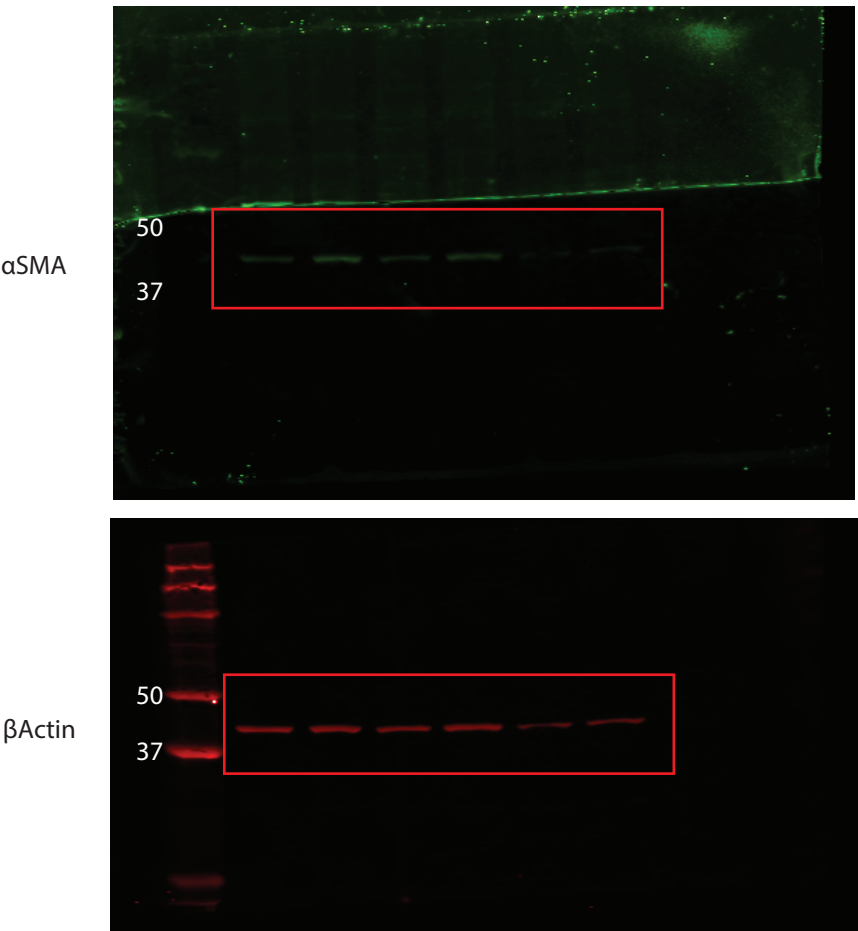

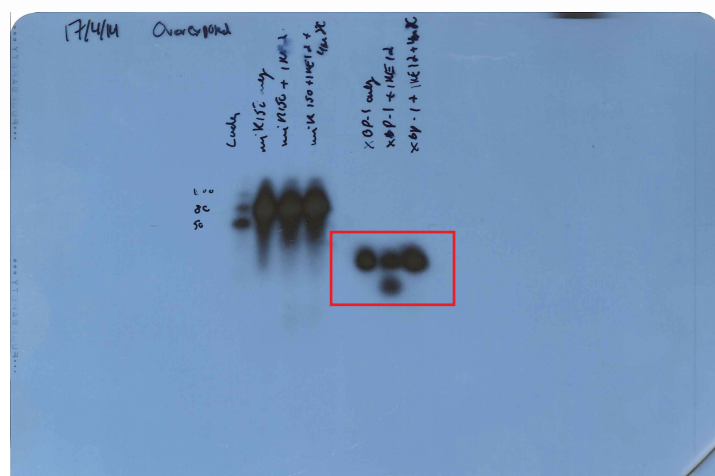

Supplement: Supplementary file 6 — Source Data for Figure 2 [file EMMM-8-729-s005.pdf]

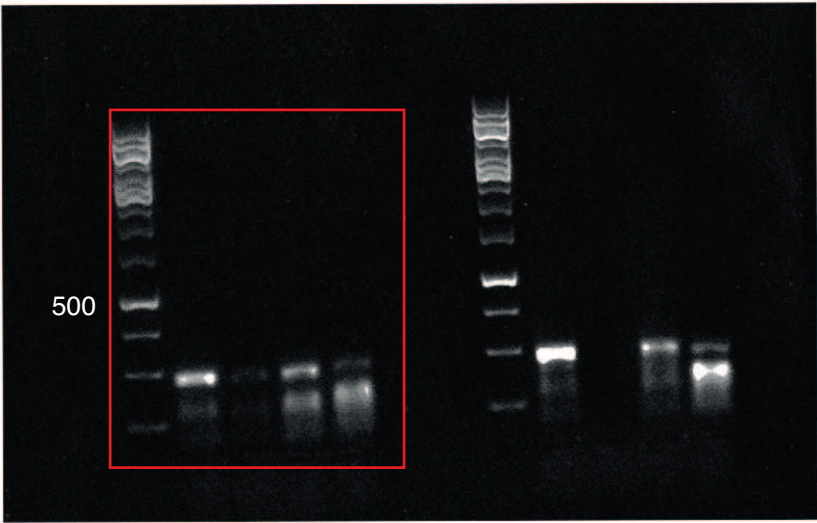

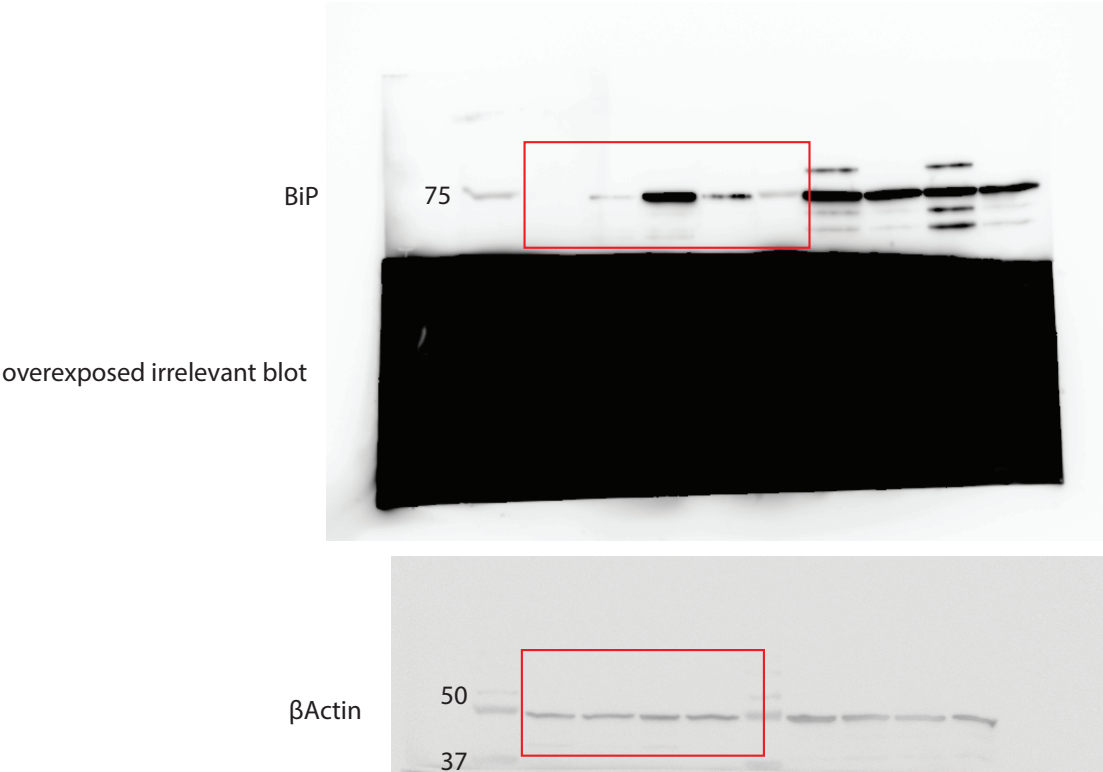

Supplement: Supplementary file 7 — Source Data for Figure 4 [file EMMM-8-729-s006.pdf]

overexposed irrelevant blot

CHOP

25

$\beta$ Actin

37

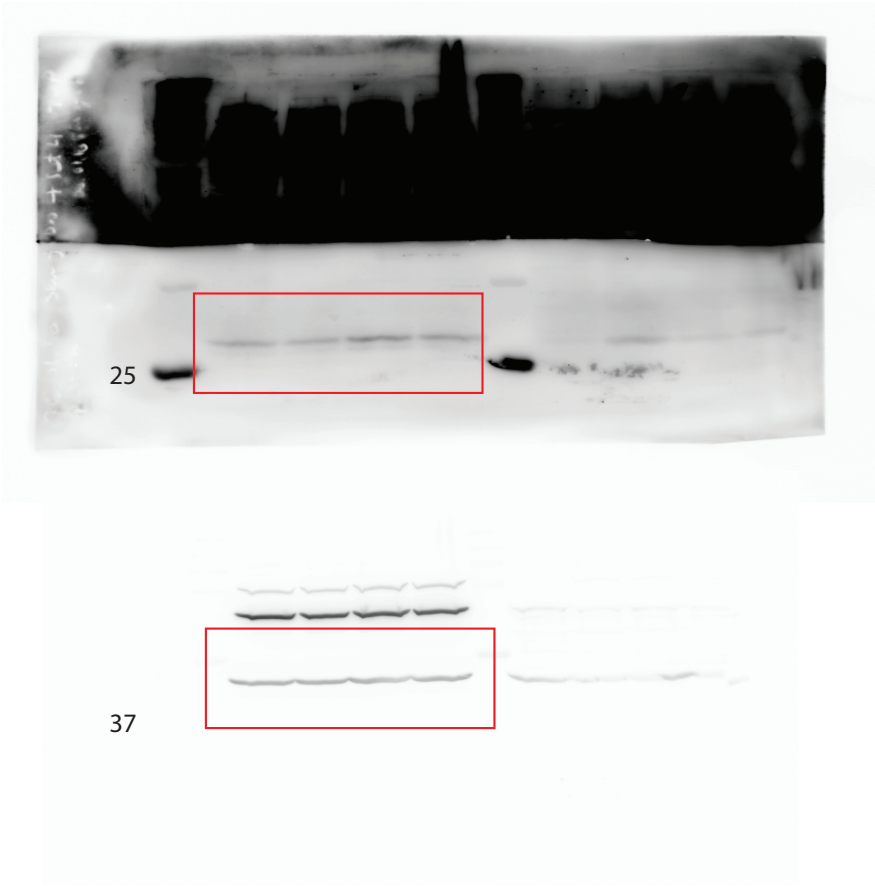

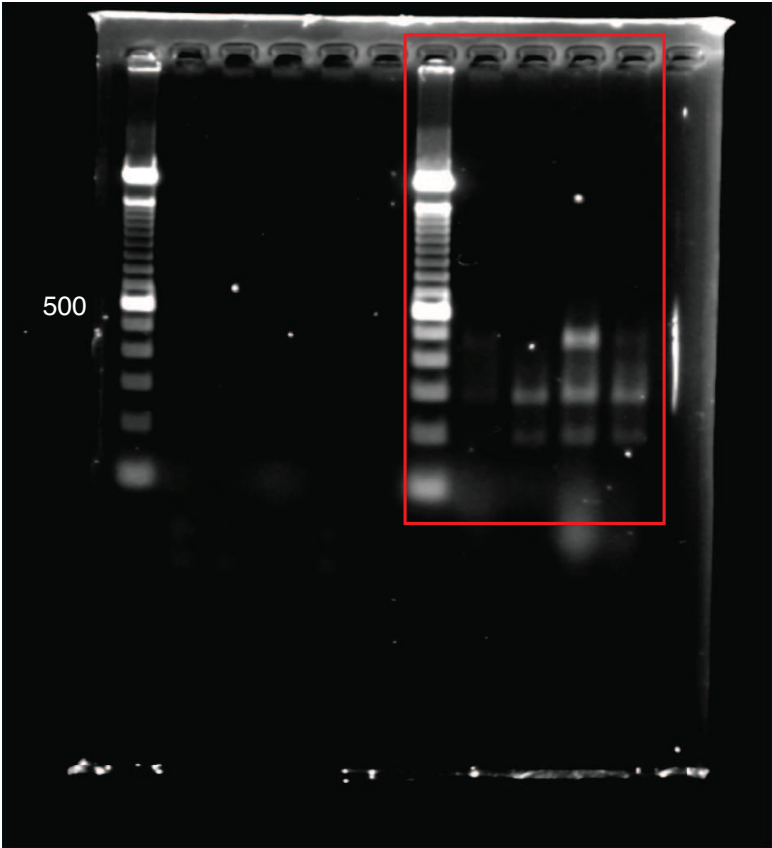

Supplement: Supplementary file 8 — Source Data for Figure 5 [file EMMM-8-729-s007.pdf]
